# Evaluation of 2D and 3D Erythroid Differentiation Protocols Using Sickle Cell Disease and Healthy Donor Induced Pluripotent Stem Cells

**DOI:** 10.3390/cells12081121

**Published:** 2023-04-10

**Authors:** Gabriele Louise Soares Martins, Carolina Kymie Vasques Nonaka, Erik Aranha Rossi, Adne Vitória Rocha de Lima, Corynne Stephanie Ahouefa Adanho, Moisés Santana Oliveira, Setondji Cocou Modeste Alexandre Yahouedehou, Clarissa Lima e Moura de Souza, Marilda de Souza Gonçalves, Bruno Diaz Paredes, Bruno Solano de Freitas Souza

**Affiliations:** 1 Gonçalo Moniz Institute, Oswaldo Cruz Foundation (FIOCRUZ), Salvador 40296-710, Brazil; 2 Center for Biotechnology and Cell Therapy (CBTC), São Rafael Hospital (HSR), Salvador 41253-190, Brazil; 3 D’Or Institute for Research and Education (IDOR), Salvador 41253-190, Brazil; 4 Hospital Universitário Professor Edgard Santos, Federal University of Bahia (UFBA), Salvador 40110-060, Brazil

**Keywords:** sickle cell disease, erythropoiesis, iPSCs

## Abstract

Background: Sickle cell disease (SCD) is a highly prevalent genetic disease caused by a point mutation in the *HBB* gene, which can lead to chronic hemolytic anemia and vaso-occlusive events. Patient-derived induced pluripotent stem cells (iPSCs) hold promise for the development of novel predictive methods for screening drugs with anti-sickling activity. In this study, we evaluated and compared the efficiency of 2D and 3D erythroid differentiation protocols using a healthy control and SCD-iPSCs. Methods: iPSCs were subjected to hematopoietic progenitor cell (HSPC) induction, erythroid progenitor cell induction, and terminal erythroid maturation. Differentiation efficiency was confirmed by flow cytometry analysis, colony-forming unit (CFU) assay, morphological analyses, and qPCR-based gene expression analyses of *HBB* and *HBG2*. Results: Both 2D and 3D differentiation protocols led to the induction of CD34^+^/CD43^+^ HSPCs. The 3D protocol showed good efficiency (>50%) and high productivity (45-fold) for HSPC induction and increased the frequency of BFU-E, CFU-E, CFU-GM, and CFU-GEMM colonies. We also produced CD71^+^/CD235a^+^ cells (>65%) with a 630-fold cell expansion relative to that at the beginning of the 3D protocol. After erythroid maturation, we observed 95% CD235a^+^/DRAQ5- enucleated cells, orthochromatic erythroblasts, and increased expression of fetal *HBG2* compared to adult *HBB*. Conclusion: A robust 3D protocol for erythroid differentiation was identified using SCD-iPSCs and comparative analyses; however, the maturation step remains challenging and requires further development.

## 1. Introduction

Sickle cell disease (SCD) is one of the most common hereditary hematological diseases worldwide, particularly affecting African descendants [1,2]. However, it remains a neglected disease with high morbidity rates, and until recently, only one disease-modifying drug, hydroxyurea, has been approved for treatment [3]. Despite being a monogenic disease caused by a missense mutation in the *HBB* gene, significant variability is observed in the degree of clinical manifestations among SCD patients due to the influence of interactions with other polymorphisms present in the individual genetic background [4]. Therefore, the field would benefit from the establishment of novel methods and models capable of providing a better understanding of pathophysiological mechanisms and drug discovery.

The advent of somatic cell reprogramming and the generation of induced pluripotent stem cells (iPSCs) has provided new perspectives for improving disease modeling and drug screening using patient cells [5,6,7,8]. Several studies, including our previous work, have described the generation and characterization of iPSCs from SCD patients [5,9,10,11,12,13,14,15]. These cells have been used in gene editing and hematopoietic differentiation studies [8,16,17,18,19,20]. Considering that most preclinical studies on SCD drug discovery have used immortalized CD34^+^ cells or animal models, the standardization of efficient hematopoietic differentiation protocols from patient-derived iPSCs would be highly beneficial [21].

In principle, iPSC hematopoietic differentiation protocols attempt to recapitulate the steps of embryonic development. Many protocols have been proposed to produce hematopoietic stem/progenitor cells (HSPCs), erythroid progenitor cells, and mature erythroid cells [22], such as by employing the use of co-culture with hematopoietic stimulating cells [23], feeder- and serum-free cultivation [16,24], or genetic modifications for efficient maturation [25,26]. Such works provide valuable directions to achieve a robust and reliable erythroid differentiation protocol, but results are often discrepant among them. The perspective of altered signaling pathways in patient cells implies that these protocols should be validated using both control and patient iPSCs. Indeed, the role of altered inflammatory pathways is now acknowledged as critical to the pathophysiology of SCD [26,27,28,29]. In the present study, we evaluated the efficiency of different protocols using 2D or 3D culture methods to establish a method of differentiating iPSCs towards the erythroid lineage using control and SCD-iPSC lines.

## 2. Materials and Methods

### 2.1. Generation and Characterization of iPSCs

This study was approved by the local Ethics Committee at São Rafael Hospital (#30875820.8.0000.0048). The iPSC lines used in this project were previously generated by reprogramming erythroblasts expanded from peripheral blood mononuclear cells (PBMCs) from three SCD patients and three healthy donors. The donors were included in the study after confirming the SCD diagnosis by hemoglobin electrophoresis and obtaining written informed consent. PBMCs were cultured in erythroblast expansion medium and transfected with episomal vectors to generate iPSCs, as previously described [9,10]. Extensive characterization of the cell lines was previously performed, including pluripotency markers, karyotype, STR analysis, mutation detection by Sanger sequencing, and β^S^ globin gene haplotype determination by polymerase chain reaction (PCR) and restriction fragment length polymorphism (RFLP) [9,10,30].

### 2.2. Culture of iPSCs

The healthy control iPSC line CBTCi003-A [10] and SCD-derived lines CBTCi005-A, CBTCi006-A, and CBTCi007-A [9] were used for the differentiation protocols. The iPSCs were grown in 6-well plates coated with Geltrex™ LDEV-Free hESC-qualified (Thermo Fisher Scientific, Waltham, MA, USA) with StemFlex™ medium (STEMCELL Technologies, Vancouver, BC, Canada), and penicillin/streptomycin 1% (Thermo Fisher Scientific), and then incubated at 37 °C in a humidified atmosphere containing 5% CO_2_. The medium was changed every 2–3 days.

### 2.3. Differentiation Step 1—Hematopoietic Stem Progenitor Cell Induction

#### 2.3.1. 2D Cultivation Method

IPSCs were dissociated by incubation with 0.05 µM EDTA solution (Thermo Fisher Scientific) for 4 min and replated at a 1:3 split ratio in StemFlex™ medium. On day 1 (D1) after passage, the medium was replaced with the defined differentiation medium adapted from Dege and Sturgeon [31]. Two different media were tested: (i) Serum-Free Medium (SFM), composed of DMEM-F12, 20% KSR, 1X Glutamax, 1X non-essential amino acids (NEAA), 82.5 nM 2-mercaptoethanol, and 1% penicillin/streptomycin (all reagents from Thermo Fisher Scientific), and (ii) StemSpan SFEM II (STEMCELL Technologies). Both media were supplemented with fibroblast growth factor (bFGF) (10 ng/mL), bone morphogenetic protein 4 (BMP4) (10 ng/mL), and vascular endothelial growth factor (VEGF) (15 ng/mL) (all from PeproTech, Cranbury, NJ, USA). On D3, the bFGF concentration was reduced to 5 ng/mL and the medium was supplemented with CHIR99021 (3 µM) (STEMCELL Technologies), which was maintained up to D4. On D6, D8, and D12, both media were supplemented with the following recombinant factors and molecules: bFGF (5 ng/mL), VEGF (15 ng/mL), erythropoetin (EPO) (2 U/mL), interleukin-6 (IL-6) (20 ng/mL), Fms Related Tyrosine Kinase 3 Ligand (FLt3L) (10 ng/mL), stem cell factor (SCF) (50 ng/mL), Insulin-like growth factor-1 (IGF-1) (40 ng/mL), interleukin-3 (IL-3) (10 ng/mL) from PeproTech and ascorbic acid (50 µg/mL), and dexamethasone (1 µM) from STEMCELL Technologies. On D12 and D16 of the protocol, cells were collected for analysis.

#### 2.3.2. 3D Cultivation Method—Embryoid Bodies (EBs)

(A.) In-house HSPC induction medium (HSPCim): after reaching 80–90% confluency, the iPSCs were dissociated into a single-cell suspension by incubation with Accutase solution (STEMCELL Technologies), followed by centrifugation at 340× *g* for 5 min and resuspension in EB-forming medium composed of DMEM-F12 supplemented with 20% of knock-out serum replacement (KSR), 1X Glutamax, 1X non-essential amino acids (NEAA), 82.5 nM 2-mercaptoethanol, and 1% penicillin/streptomycin (Thermo Fisher Scientific). The following recombinant factors were added to the medium: bFGF (4 ng/mL), VEGF (20 ng/mL), SCF (40 ng/mL), and BMP4 (20 ng/mL). The Rho pathway inhibitor Y-27632 (10 µM) (STEMCELL Technologies) was also added to the medium. The cells were counted and plated for EB formation based on aggregations of 6000 cells. Three methods for cell aggregation were tested based on previously described protocols [32,33,34,35,36] or the manufacturer’s instructions: non-adherent U-bottom 96 well plate (ULA; Thermo Fisher Scientific), hanging drop (Hdrop), and Aggrewell™ 800 plate (Aggw; STEMCELL Technologies). On D4, the EBs were transferred to a non-adherent Petri dish containing fresh medium without Y-27632. On D8 and D12, the medium was changed. On D12 and D16, cells from the supernatant were collected for analysis.

(B.) Commercial medium: STEMdiff™ APEL™2 Medium (APEL; STEMCELL Technologies) was used for EB formation based on the protocol of Ng and collaborators [34]. The medium was supplemented with BMP4 (10 ng/mL), bFGF (10 ng/mL), SCF (50 ng/mL), VEGF (10 ng/mL), penicillin/streptomycin (1%), and Y-27632 (10 µM). After dissociation and counting, the iPSCs were resuspended in this medium and plated for aggregation on ULA or Aggw plates. In both cases, each EB was formed from 3000 starting iPSCs. On D6, 50 µL fresh medium was added to each ULA well. For Aggw, 750 µL of the medium was removed and the same volume of fresh medium was added. On D9, the EBs were transferred to non-adherent straight-bottomed 24-well plates at a proportion of 100 µL per transferred EB, and fresh medium was added. On D12 and D16, the cells in the supernatant were collected for analysis.

### 2.4. Differentiation Step 2—HSCP towards Erythroid Progenitor Cells (EPCs)

Samples from the end of Step 1 were collected and incubated with a medium for erythroid specification and expansion. Two different recombinant factor cocktails were tested using complete StemPro34 (SP34) (Thermo Fisher Scientific) as basal media: (i) SP34 supplemented with four factors (SP34+4F): holo-transferrin (500 µg/mL; R&D Systems, Minneapolis, MN, USA), SCF (50 ng/mL), EPO (2 U/mL), and IL-3 (10 ng/m); and (ii) SP34 supplemented with six factors (SP34+6F): SCF (50 ng/mL), VEGF (50 ng/mL), IL-6 (30 ng/mL), IL-3 (30 ng/mL), thrombopoietin (TPO) (30 ng/mL/mL) (PeproTech), and EPO (3 U/mL). Both media were supplemented with 1% penicillin/streptomycin solution. SP34+4F medium was used only for cells in suspension. SP34+6F medium was adapted from the protocol of Ng et al. [34] and used for the adherent culture of EBs in 24-well plates coated with Geltrex. Under all conditions, the medium was changed every 3–4 days until D26, when the cells in the supernatant were collected for analysis.

### 2.5. Differentiation Step 3—Terminal Maturation of Erythroid Cells

The cells in the culture supernatant were collected at the end of step 2 and incubated with the specific medium for final erythroid maturation, based on the protocol by Huang et al. [6], composed of Iscove’s Modified Dulbecco’s Medium (IMDM) (Thermo Fisher Scientific) supplemented with EPO (3 U/mL), holo-transferrin (500 µg/mL), heparin (4 U/mL), and insulin (10 µg/mL) (Sigma-Aldrich, St. Louis, MI, USA). The medium was changed every 2–3 days, and the cells were counted for volume adjustment and passaging, targeting a maximum cell density of 4 × 10^6^ cells/mL. At the end of this stage (D33), cells were collected for analysis.

### 2.6. Flow Cytometry

To immunophenotype the differentiating cells, the samples were dissociated, centrifuged 340× *g* for 5 min, and resuspended in 1X PBS. Antibodies were added to the cell suspension and incubated for 20 min at 21 °C. Anti-human antibodies CD34-PE (1:200) and CD43-BB515 (1:400) (BD Biosciences, San Jose, CA, USA) were used to characterize the HSPCs. CD235a-PE (1:400; Beckman Coulter, Indianapolis, IN, USA) and CD71-FITC (1:500; BD Biosciences) were used to label the EPCs at the end of Step 2. For mature erythroid cells, the samples were stained with 5 µM DRAQ5 DNA dye (Thermo Fisher Scientific) and CD235a antibody. The samples were then washed with PBS 1X. At least 10,000 events were acquired using a flow cytometer (LSR Fortessa, BD Biosciences). Data were analyzed using Kaluza v2.2 (Beckman Coulter) and FlowJo v10 (BD Biosciences) analysis software. Hoechst 33342 (Thermo Fisher Scientific) was used to select live cells, and the isotype controls were mouse IgG1 (κ-BB515), mouse IgG1 (κ-PE), and mouse IgG2a (FITC) (BD Biosciences).

### 2.7. Colony Forming Unit (CFU) Assay

MethoCult (STEMCELL Technologies) was used to assess the functional capacity of HSPCs following the manufacturer’s instructions. Briefly, 5000 cells were collected based on the percentage of CD34^+^ cells in the samples from D16 of Step 1 and then added to 3 mL of MethoCult. After homogenization, 1.1 mL was placed in a 35 mm culture plate in duplicate and incubated for 14 days. CFUs were registered using an inverted microscope (Nikon Eclipse Ti-U, Nikon Instruments Inc., Melville, NY, USA) for the identification of colony units and quantification.

### 2.8. Morphological Evaluation

Cells at different stages of differentiation were collected, washed with 1X PBS, and centrifuged (23× *g* for 5 min, acceleration medium, 21 °C) on a glass slide using a Shandon CytoSpin 4 Cytocentrifuge (Thermo Fisher Scientific). The slides were stained with Giemsa and analyzed under a microscope (Nikon Eclipse Ti-U) at 400× or 1000×.

### 2.9. Analysis of Hemoglobin Expression by RT-qPCR

Total RNA from differentiated cells was extracted using the TRIzol chloroform method, followed by precipitation with isopropanol. The samples were quantified using a NanoDrop 8000 spectrophotometer (Thermo Fisher Scientific). cDNA was produced from the reverse transcription of 0.5 µg RNA using a high-capacity cDNA Reverse Transcription kit (Thermo Fisher Scientific), according to the manufacturer’s recommendations. RT-qPCR reactions were performed in triplicate using the endogenous normalizing gene GAPDH (Hs99999905_m1), in addition to specific probes for beta globin (Hs00747223_g1 *HBB*) and gamma globin (Hs00361131_g1 HBG2). For the reactions, TaqMan Universal PCR MasterMix (Thermo Fisher Scientific) was used under the following cycling conditions: 50 °C 20 s, 95 °C 10 min, 95 °C 15 s, 60 °C 1 min (40×). The equipment used was ABI 7500 FAST (Thermo Fisher Scientific).

### 2.10. Investigation of β^S^-Globin Gene Cluster Haplotypes

Molecular analyses were carried out on genomic DNA extracted from iPSCs using the Flexigen 250 kit (Qiagen, Hilden, Germany). Beta S (β^S^) globin gene cluster haplotypes were investigated using the polymerase chain reaction-restriction fragment length polymorphism (PCR-RFLP) method [37]. Briefly, PCR amplification was performed using five primer pairs (3/4, 5/6, 6/7, 8/9, and 10/11). The reaction mixture contained genomic DNA, primer (25 pmol/μL), dNTPs (2 mM) (Thermo Fisher Scientific), Taq polymerase (5U/μL), MgCl_2_ (50 mM), buffer 10X (Taq DNA Polymerase Kit, Thermo Fisher Scientific), and a sufficient volume of free DNase H_2_O for 50 μL. PCR was performed at 94 °C for 10 min for initial denaturation, followed by 35 cycles of denaturation at 94 °C for 45 s, annealing at a specific temperature (Table 1) for 45 s and an extension at 72 °C for 90 s, and a final extension at 72 °C for 10 min. Amplicon (20 μL) was digested with the restriction enzymes *Xmn*I, *Hinc*II, *Hind*III, or *Hinf*I (Thermo Fisher Scientific). Buffer 10X and a suitable amount of PCR H_2_O were added to this mixture. Regarding the reaction containing *Xmn*I, bovine serum albumin was added. Amplicons and digested DNA fragments were electrophoresed on 1% and 2% agarose gels (Thermo Fisher Scientific) for 1–2 h, respectively, and visualized under ultraviolet light (Table 1).

### 2.11. Relevant Reagents and Statistical Analysis

The relevant commercial basal media, growth factors, cytokines, isotypes, and specific primary antibodies are listed in Table S1.

For the statistical analysis, parametric data were analyzed using unpaired t-tests, and standard errors of the mean were plotted on a graph. The results were considered significant at *p* < 0.05. mRNA expression was calculated by the threshold method [38] using log 2 (fold change) values obtained through the calculation of 2^−ΔΔCt^. Data were obtained in triplicate and evaluated by ANOVA using the Bonferroni post-test with the aid of Prism v7 software (GraphPad, Boston, MA, USA). The results were considered significant at *p* < 0.05 (unadjusted *p*-value).

## 3. Results

### 3.1. Induction of iPSCs towards HSPCs (Step 1)

**Figure 1:**
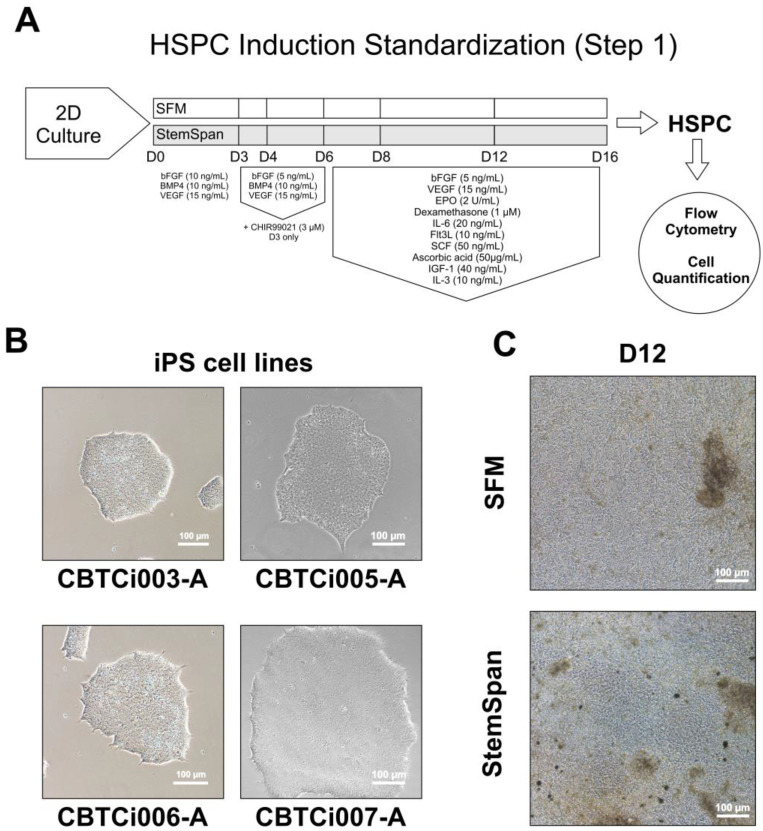
Workflow of the Hematopoietic Stem Progenitor Cell (HSPC) induction methods (Step 1), using different 2D and 3D culture methods, basal media, and supplements (**A**). Representative images of Induced Pluripotent Stem Cell (iPSC) lines CBTCi003-A, CBTCi005-A, CBTCi006-A, and CBTCi007-A at D0 (**B**). Representative images of 2D culture induction at D12 using SFM and StemSpan media with factors (**C**). SFM: Serum-Free Medium.

The iPSC lines (n = 4) included in the experiments and their respective genotypes are listed in Table 2. Initially, we tested different conditions using a 2D cultivation method (Figure 1A) with the intention of developing a robust, cost-effective, and faster protocol. At the beginning of the protocol, the iPSCs on D0 showed compact colony formation (Figure 1B). After incubation with either SFM or StemSpan supplemented with growth factors, we observed expansion and morphological alterations of the adhered cells in culture (Figure 1C). Flow cytometry analysis of the cell suspension revealed a very low percentage of CD34^+^/CD43^+^ cells (Figure S1).

**Figure 2:**
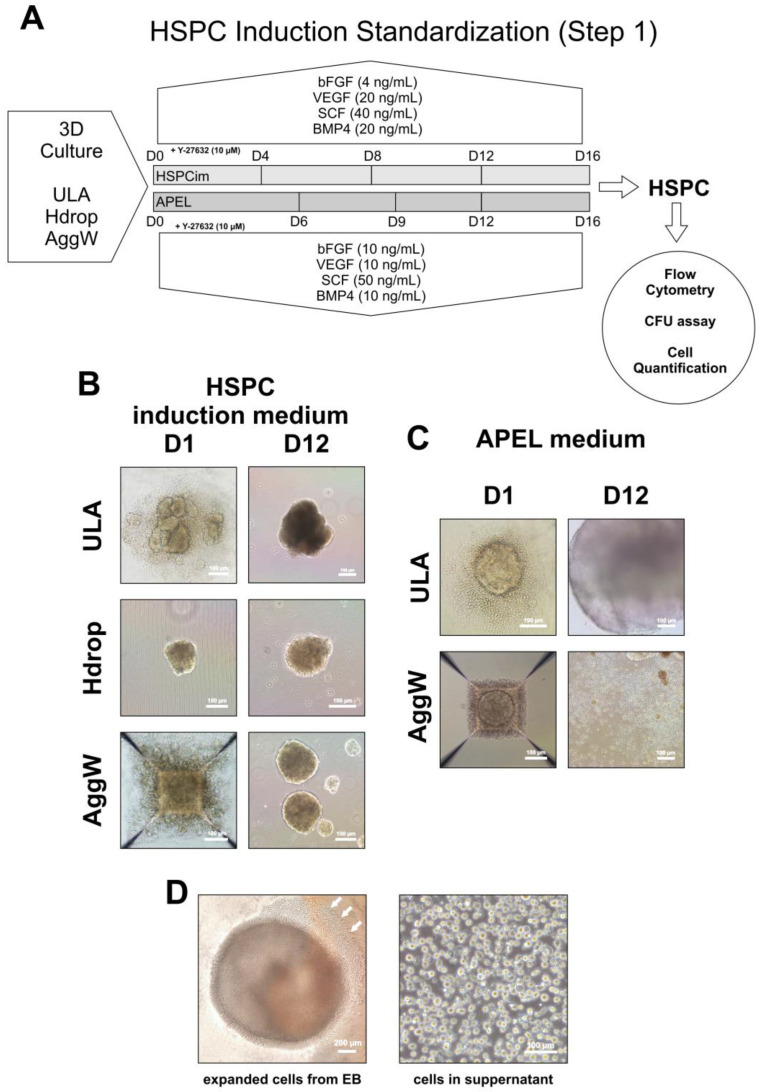
Workflow of the Hematopoietic Stem Progenitor Cell (HSPC) induction methods (Step 1), using different 3D culture methods, basal media, and supplements (**A**). Representative images of EBs formed on D1 and D12 using HSPCim in three different aggregation methods (**B**). Representative images of EBs formed on D1 and D12 using APEL in two different aggregation methods (**C**). Representative image of cells sprouting (white arrows) from an EB and expansion in the supernatant (**D**). HSPCim: Hematopoietic Stem/Progenitor Cell induction medium.

For subsequent assays, we compared different erythroid differentiation protocols using 3D culture techniques based on previously reported methods [6] (Figure 2A). We compared three cell aggregation methods (ULA, Hdrop, and Aggw) using HSPCim with factors and observed similar EB sizes and morphologies on D12 for all aggregation methods (Figure 2B). This finding showed that these methods have the capacity to aggregate iPSCs and form 3D structures. Although all the methods were efficient, the Hdrop process was not performed in the following tests because it is a labor-intensive method. For the APEL media test assay, we used the ULA and AggW aggregation methods and observed that self-assembly occurred with lower numbers of non-aggregated dispersed cells surrounding the EB on D1 when compared to HSPCim (Figure 2C). At D12, the ULA EBs from both cell lines were larger than those obtained using HSPC induction media. In contrast, the AggW EBs from both cell lines underwent spontaneous disaggregation (Figure 2C). We also observed the presence of cells sprouting from the EB and expansion in the suspension (Figure 2D). Based on these observations, new differentiation experiments were performed using ULA cell aggregates and APEL medium supplemented with the described growth factors.

**Figure 3:**
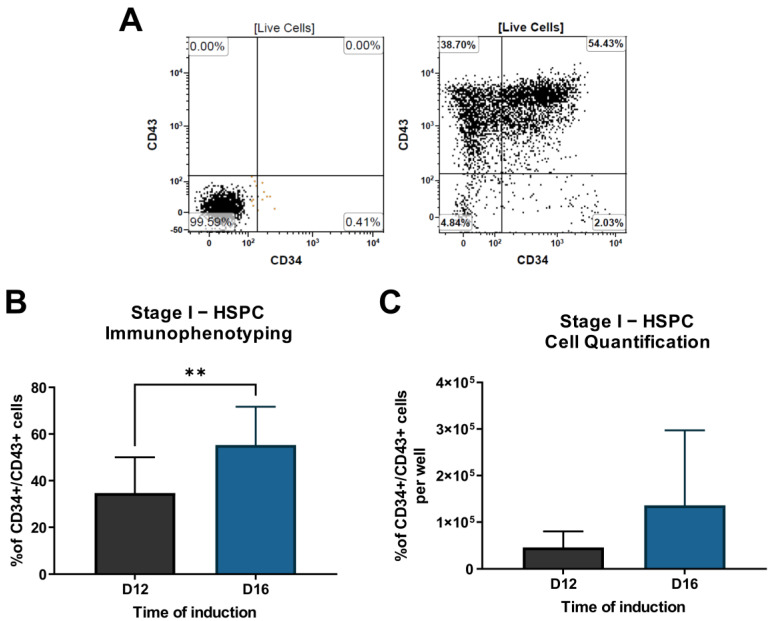
Representative flow cytometry dot plots analysis of CD34 and CD43 markers for HSPC of D12 and D16 samples (left: isotype controls; right: specific antibodies) (**A**). Percentage of cells (**B**) and cell quantification per well (**C**) of CD34^+^/CD43^+^ subpopulation at D12 and D16. ** *p* = 0.0031.

To evaluate the presence of HSPCs in the samples, immunophenotyping was performed using flow cytometry. All cell lines successfully differentiated into CD34^+^/CD43^+^ HSPCs. Our results showed a 34.7% ± 15.3% HSPC subpopulation on D12, and a significant percentage increase (*p* < 0.0031) was observed on D16, with the HSPC subpopulation representing 55.3% ± 16.4% (Figure 3B). Although HSPC subpopulations increased in percentage, CD34^+^/CD43^+^ cell quantification showed a non-statistically significant increasing trend from D12 (4.6 ± 1.1 × 10^4^ cells) to D16 (13.6 ± 4.6 ×10^4^ cells) (Figure 3C). Considering the initial number of cells on D0 and D16, we obtained an approximately 45-fold expansion of HSPCs.

**Figure 4:**
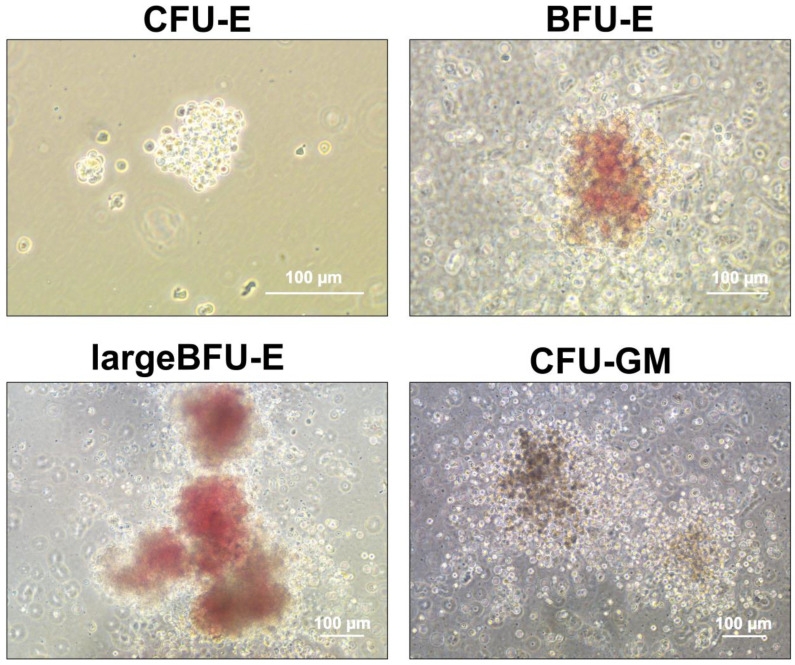
Representative images of Colony Forming Unit (CFU) assay from iPSC-derived HSCs.

The hematopoietic functional test of the HSPCs produced on D16 showed colonies that were identified as erythroid progenitor cells, including CFU-E, BFU-E, large BFU-E, granulocyte and/or macrophage progenitor cells, and CFU-GM (Figure 4). The percentage range of observed colonies was 46 ± 11% for BFU-E (regular and large), 21 ± 6.1% for CFU-E, 32 ± 6.7% for CFU-GM, and 0.95% ± 1.9% for CFU-GEMM.

### 3.2. Induction of HSPC towards EPCs (Step 2)

**Figure 5:**
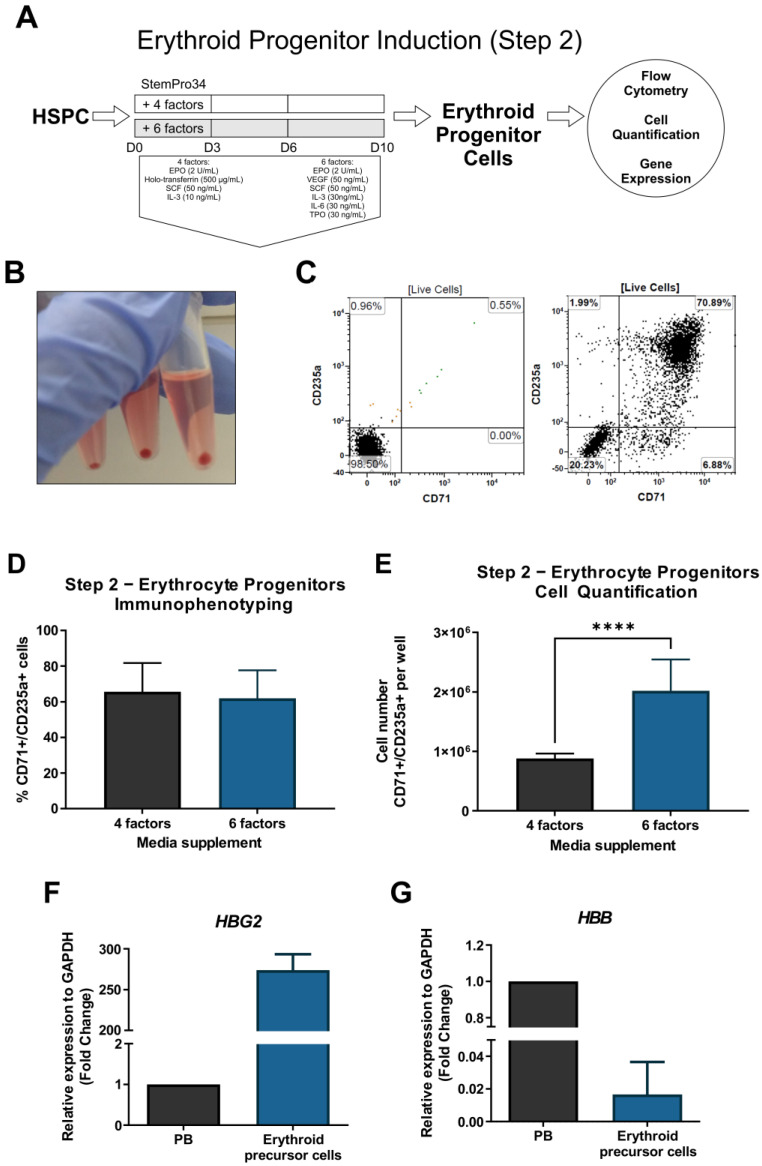
Workflow of Step 2 and analysis of iPS-derived erythroid progenitor cells (EPCs) (**A**). Representative image of red cell pellet observed after centrifugation of Step 2 D10 samples (**B**). Representative flow cytometry dot plot analysis of CD71 and CD235a as markers for EPCs (left: isotype control; right: specific antibodies)(**C**), percentage of cells (**D**) and cell quantification (**E**) of the CD71^+^/CD235a^+^ subpopulation in SP34+4F and SP34+6F media. Gene expression analysis of *HBG2* gene (**F**) and *HBB* gene (**G**) compared to peripheral blood (PB). **** *p* < 0.0001.

In Step 2, we compared the production of EPCs promoted by the SP34+4F and SP43+6F media (Figure 5A). Samples from D10 of Step 2 were analyzed using CD71 and CD235a antibodies as EPC markers. The collected samples showed vivid red pellets after centrifugation (Figure 5B). Immunophenotype analysis showed the presence of CD71^+^/CD235a^+^ cells in the samples (Figure 5C), although significant differences were not observed in this subpopulation cultivated with SP34+4F media (65.7% ± 16%) and SP34+6F media (62.0% ± 15.6%) (Figure 5D). Meanwhile, the quantification of CD71^+^/CD235a^+^ cells showed a significant increase (*p* < 0.0001) when using SP34+6F (8.81 ± 8.3 × 10^5^ cells) compared to SP34+4F (2.02 ± 5.2 × 10^6^ cells) (Figure 5E). On D16 in Step 1 and D10 in Step 2, we obtained a 14-fold expansion of erythroid precursor cells. We also analyzed globin gene expression in total cells and observed a high expression of *HBG2* compared to that in peripheral blood (PB) (Figure 5F), showing that the produced cells present a fetal phenotype. In contrast, the samples showed low *HBB* gene expression (Figure 5G).

### 3.3. Maturation of Erythroid Cells (Step 3)

**Figure 6:**
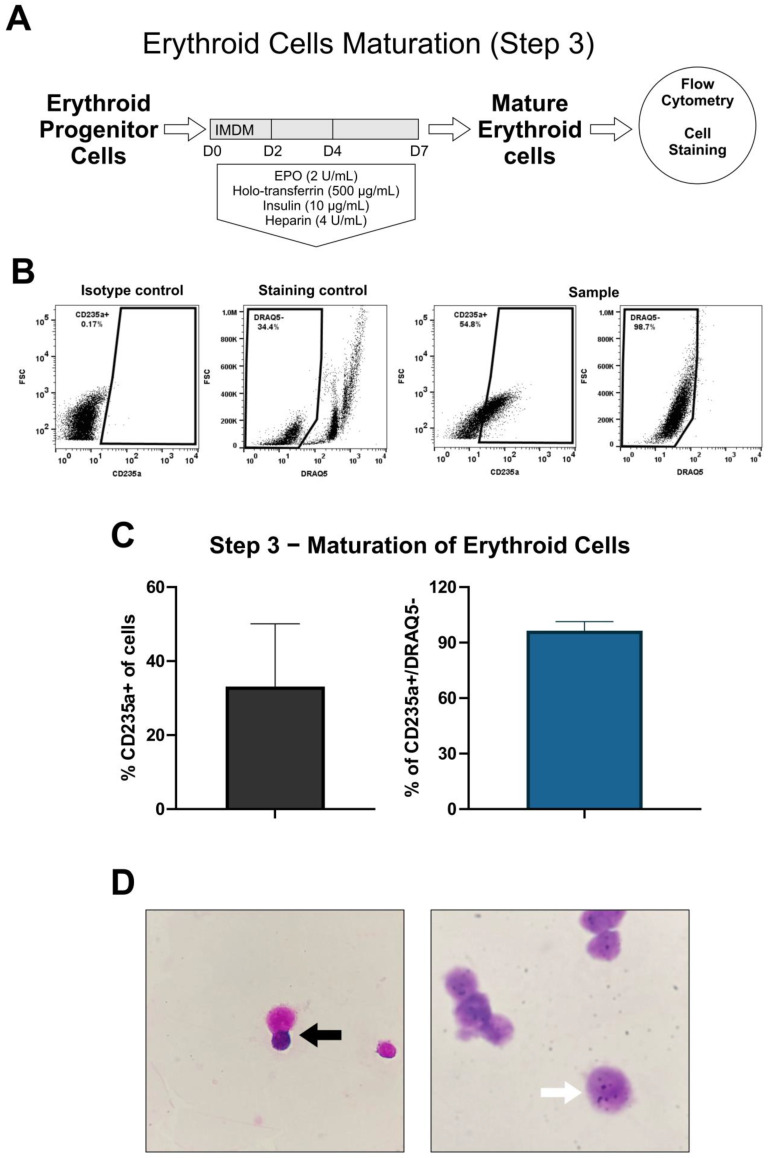
Workflow of the erythroid maturation induction (Step 3) method and analysis EPC (**A**). Representative flow cytometry dot plot analysis of CD235a and DRAQ5 for mature erythroid cells (far left: isotype control; left: DRAQ5 staining of control PBMC; right: CD235a specific antibody; far right: DRAQ5 on sample) (**B**). Percentage of CD235a^+^ (left) and CD235a^+^/DRAQ5- (right) at D7 of step 3 (**C**). Representative images of mature erythroid cells. Cell enucleation (black arrow) and reticulocyte-like cells (white arrow) were observed under optical microscopy, by Giemsa staining. Magnification: 400× (left) and 1000× (right) (**D**).

Step 3 was performed by culturing the cells in suspension in maturation medium for another 7 days to generate mature erythroid cells (Figure 6A). Flow cytometry analysis (Figure 6B) showed a CD235a subpopulation (33% ± 17%), in which DRAQ5- cells accounted for 96% ± 4.9% (Figure 6C), suggesting that CD235a^+^ erythroid cells underwent enucleation, indicating erythroid maturation. Morphological evaluation of the stained cells allowed us to identify orthochromatic erythroblasts at the beginning of the maturation step and reticulocyte-like cells at the end of the assay (Figure 6D).

## 4. Discussion

Several studies have demonstrated that iPSCs can be used to obtain erythroid cells based on either EBs (3D culture) [35,36,39] or monolayers (2D culture) [24,40,41]. In the present study, we present a comparative analysis of different protocols to generate hematopoietic progenitor cells and erythroid cells from iPSCs obtained from healthy donors and SCD-iPSCs. Due to the small sample size, our data does not allow for comparison between healthy cells and SCD-iPSCs, but it contributes to the evaluation of the reproducibility of published reports, and it helps define the most appropriate methodology for obtaining erythroid cells from iPSCs, which is necessary before these models can be successfully applied in drug screening and disease-modeling studies. Indeed, the importance of validation studies is demonstrated by differences in the efficiency of human iPSC differentiation protocols, which may be influenced by several factors, including inter-laboratory and cell line variability.

The first step in differentiation is to obtain HSPCs, which are defined by the co-expression of CD34 and CD43 [23] that characterize primitive hematopoietic progenitors [42]. Although we did not achieve satisfactory results using the 2D culture methodology, other authors have achieved in vitro erythropoiesis through monolayer culture. Nonetheless, most of the reported 2D protocols involve coculture with OP9 cells [42,43], which were avoided here because of the goal of establishing xeno-free protocols.

Cellular interactions in the microenvironment are critical for proper hematopoietic differentiation [44,45,46,47]. Therefore, by enhancing cell interactions, 3D models based on EB differentiation can provide a theoretical advantage over 2D monolayer models. Data from different studies [48] show that within the formed 3D structure, the molecular and morphological events resemble the beginning of human embryonic development [49] and favor the induction of hematopoietic lineages [6,35,50]. Our data support the more efficient generation of HSPCs and increased cell expansion using 3D methods, and these results could be further scaled up. Among the different methods used for EB formation, the hanging drop method has been widely applied in research on mouse pluripotent stem cells, with few successful reports in human cell studies. As a labor-intensive method, other aggregation methods have been shown to be more efficient, corroborating the data described herein [51,52,53]. We also observed that Aggw plate and APEL medium presented EB deterioration along the hematopoietic differentiation. In our experiments, we utilized the same media and cell numbers for EB formation for the Aggw and ULA methods, and although both methods were able to initially form EBs with similar size and characteristics, an important difference was observed in the steps following aggregation. It was not in our scope to evaluate the mechanisms involved in that event, but this observation ended up favoring the ULA plates aggregation method. It is known that large EBs may present areas of core necrosis due to low oxygen/nutrient diffusion, and EBs that are too small may not survive or perform inefficiently in differentiation protocols [54]. EB viability and the yield in terminal differentiation may vary in a size-dependent manner. Our results highlight the importance of choosing and validating an aggregation method that better suits the desired applications. For instance, different results in terms of EB survival and differentiation performance have been reported for EB formed even for V and U bottom plates [55].

After differentiation Step 1, we achieved an average induction efficiency of 55% for iPSCs towards CD34^+^/CD43^+^ HSPCs. Kessel et al. [56] obtained an induction efficiency of approximately 10% for EBs towards CD34^+^/CD43^+^ HSPCs using a similar protocol. In addition, we generated HSPCs in high quantities when using the APEL medium, which allowed for the continuation of differentiation Steps 2 and 3. These data corroborate the study published by Reis et al. [11] concerning the use of SCD-derived iPSCs to perform hematopoietic differentiation with EB formation, including the use of APEL medium and the emergence of surrounding cells from EB, as previously mentioned. In contrast, our work showed an increase in the percentage of CD34^+^/CD43^+^ cells using the methodology described herein, while Reis et al. observed a reduction or loss in the percentage of these hematopoietic markers. In addition to the already known epigenetic memory effect over iPSCs [57,58], the observed discrepancy may be explained by the intrinsic cell line variability, such as alterations in the expression levels of hematopoietic-related genes and/or other gene variants that can alter cell behavior to the same stimuli [59].

Our Step 1 cell quantification comparison of D12 and D16 suggests that increasing the time of induction delivers a higher number of HSPCs, thus allowing robust HSPC expansion, which is useful for detailed analysis of hematopoietic development and disease modeling. Concerning the number of erythroid progenitor cells at D10 of Step 2, we observed a 14-fold expansion of CD71^+^/CD235a^+^ from HSPCs, which resulted in a 630-fold cell expansion related to that at the beginning of the 3D protocol. Other authors have shown an expansion of approximately 10-fold in erythroblasts derived from different sources of iPSCs using a co-culture with the OP9 cell line [60]. Deng et al. obtained a 530-fold expansion at the end of the erythroblast phase using platelet lysate in a medium [61]. Although these differences can be associated with the cultivation method, they serve as references to demonstrate the enforceability of our protocol. Another study by the above-cited group [16] also evaluated the functional activity of HSPCs by CFU assay and identified the formation of colonies of macrophages, granulocytes, and erythrocytes. Our work corroborates these findings, which demonstrate that the functional abilities of healthy donors and sickle cell iPSC-derived HPSCs are similar and able to generate hematopoietic colonies.

After cultivation with specific factors for erythroid differentiation and expansion of erythroblasts, in Step 2, we obtained approximately 65% CD71^+^/CD235a cells. The yield of cells obtained at the end of stage II was 34- and 42-times the number of the initial cells depending on the analyzed strain, and these values are close to those obtained by Huang and collaborators for the same stage [6]. However, other studies that used EB formation to obtain erythroid cells reported the presence of more than 80% of cells labeled for either CD71 or CD235a [36,62]. The red cell pellet suggests that the induced cells produced hemoglobin. The gene expression of *HBG2* and *HBB* in iPSC-derived erythroid progenitor cells suggests that differentiated cells have a fetal phenotype and are not capable of switching to a mature phase. Other studies have reported the same profile [23,42,62]. Maturation and hemoglobin switching are critical steps for hematopoietic disease modeling, especially in SCD cell lines, and these steps should be optimized in future studies so that a disease phenotype (i.e., sickling) can be reproduced in vitro. In this sense, alternative protocols that show highly efficient production of hemoglobin β using different approaches, such as immortalization of erythroid precursor cells and modulation of *BCL11A* expression [25,26,63], in vivo maturation in NOD/SCID mice [64], or using cells treated with human platelet lysate [63], should be further assessed. Recently, Chen et al. reported that erythroid terminal differentiation was improved in a culture of HSPCs derived from human embryonic stem cells by adding an aryl hydrocarbon receptor antagonist [65], and this represents a less laborious approach that is compatible with SCD disease models.

## 5. Conclusions

In this study, we describe the application of iPSC lines derived from healthy donors or SCD individuals to obtain erythroid cells. By optimizing protocols described in the literature and different methodologies, a protocol was developed for obtaining erythroid cells from iPSCs that express specific markers and have typical morphological characteristics; however, the cells present a fetal phenotype, requiring further efforts to improve maturation. The results of this study will serve as a basis for the development of 3D protocols and may contribute to establishing a platform for screening drugs for sickle cell anemia, as well as studying the mechanisms involved in the disease and developing new therapies.

## Figures and Tables

**Table 1:** Primers and PCR-RFLP conditions for β^S^ globin gene cluster haplotypes.

Gene	Primer	Amplicon (pb)	Digestion Product	Annealing Temperature	Restriction Enzime
5′γ^G^	3 and 4	650	450 + 200	57 °C	*Xmn*I *
γ^G/^γ^A^	5 and 6	780	440 + 340	60 °C	*Hind*III
γ^G^/γ^A^	6 and 7	760	360 + 400	62 °C	*Hind*III
Ψβ	8 and 9	700	360 + 340	60 °C	*Hinc*II
3′ψβ	10 and 11	590	470 + 120	57 °C	*Hinc*II

* Add Bovine Serum Album.

**Table 2:** iPSC cell line and haplotype identification.

iPSC Line	Phenotype	β^S^ Haplotypes
CBTCi003-A	Healthy (HbAA)	Not evaluated
CBTCi005-A	Sickle cell anemia (HbSS)	Benin/CAR
CBTCi006-A	Sickle cell anemia (HbSS)	Benin/Benin
CBTCi007-A	Sickle cell anemia (HbSS)	Benin/CAR

CAR: Central Africa Republic.

## Data Availability

All relevant data is available from the authors upon reasonable request.

## Supplementary Materials

The following supporting information can be downloaded at: https://www.mdpi.com/article/10.3390/cells12081121/s1, Table S1: List of relevant commercial reagents; Figure S1: Flow cytometry analysis of 2D erythroid differentiation method.
