# Supplementary material for: Evaluation of 2D and 3D Erythroid Differentiation Protocols Using Sickle Cell Disease and Healthy Donor Induced Pluripotent Stem Cells
Source: Cells. 2023 Apr 10;12(8):1121. doi: 10.3390/cells12081121 (PMC10137038; doi:10.3390/cells12081121)
Supplement: Supplementary file 1 [file cells-12-01121-s001.zip › cells-1915079-supplementary.pdf]

Table S1: Brand and catalog number of relevant reagents (basal media, growth factors, cytokines, and antibodies)

Reagent	Brand	cat. number
Ascorbic Acid	STEMCELL Technologies	72132
bFGF	PeproTech	100-18B
BMP-4	PeproTech	120-05
CD34-PE	BD Biosciences	555822
CD43-BB515	BD Biosciences	564542
CD71-FITC	BD Biosciences	340717
CD235a-PE	Beckman Coulter	A07792
Dexamethasone	STEMCELL Technologies	72092
DRAQ5	Thermo Fisher Scientific	62251
Flt3L	PeproTech	300-19
EPO	PeproTech	100-64
Heparin	Sigma-Aldrich	H3149
Holo-transferrin	R&D Systems	2914-HT
IGF-1	PeproTech	100-11
IL-3	PeproTech	200-03
IL-6	PeproTech	200-06
Insulin	Sigma-Aldrich	91077C
MethoCult™ H4034 Optimum	STEMCELL Technologies	04034
Mouse IgG1,κ -BB515 isotype control	BD Biosciences	564416
Mouse IgG1,K -PE isotype control	BD Biosciences	555749
Mouse IgG2a-FITC isotype control	BD Biosciences	340764
SCF	PeproTech	300-07
STEMdiff™ APEL™2 Medium	STEMCELL Technologies	05270
StemPro™-34 SFM	Thermo Fisher Scientific	10639011
TPO	PeproTech	300-18

VEGF	PeproTech	100-20
Y-27632 (Dihydrochloride)	STEMCELL Technologies	72304
Taq DNA Polymerase Kit	Thermo Fisher Scientific	43680

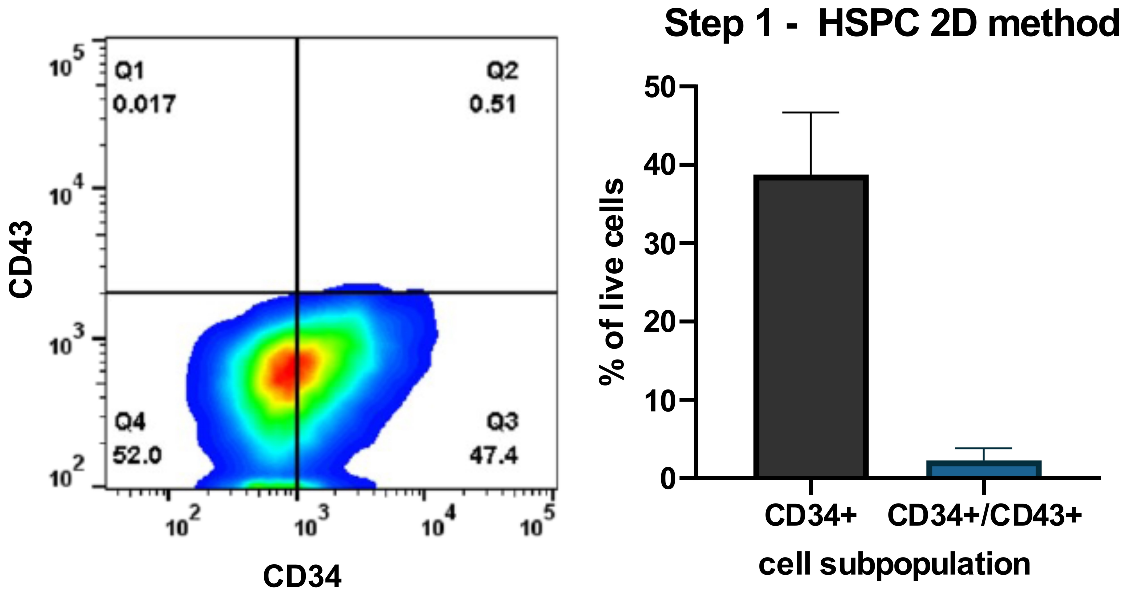

Figure S1: Flow cytometry analysis of Step 1 hematopoietic differentiation using 2D culture method.
